# Meningococcemia in a patient coinfected with hepatitis C virus and HIV.

**DOI:** 10.3201/eid0606.000615

**Published:** 2000

**Authors:** C G Nelson, M A Iler, C W Woods, J A Bartlett, V G Fowler

**Affiliations:** Duke University Medical Center, Durham, North Carolina 27710, USA.

## Abstract

We describe the first reported case of meningococcemia in a patient coinfected with hepatitis C virus and HIV. Hypocomplementemia secondary to hepatic dysfunction may have enhanced the patient's susceptibility to meningococcal infection.


### Meningococcemia in a Patient Coinfected with Hepatitis C Virus and HIV

**Christopher G. Nelson, Mark A. Iler, Christopher W. Woods, John A. Bartlett, and Vance G. Fowler, Jr.**
Duke University Medical Center, Durham, North Carolina, USA

We describe the first reported case of meningococcemia in a patient coinfected with hepatitis C virus and HIV. Hypocomplementemia secondary to hepatic dysfunction may have enhanced the patient's susceptibility to meningococcal infection.

Coinfection with hepatitis C virus (HCV) and HIV is an emerging public health problem. While coinfection with HIV can accelerate the progression of HCV (1,2), the impact of dual infection on other infectious diseases is unknown. We describe the first reported case of meningococcal infection in a patient coinfected with HCV and HIV.

### Case Report

A 45-year-old woman was admitted to our hospital emergency department in September 1999 with influenza-like symptoms (36 hours) and fever, headache, and myalgias (12 hours). The patient's medical history included ongoing injection drug use, coinfection with HIV and HCV, an episode of *Staphylocccus aureus* cervical osteomyelitis, three culture-confirmed episodes of *Streptococcus pneumoniae* pneumonia, and HIV-associated thrombocytopenia. Her most recent CD4 count was 149 cells/µL, and in April 1999, her plasma HIV RNA level was 2,000 viral copies/ µL. She had received pneumococcal polysaccharide vaccine in 1996. Because of ongoing injection drug use, she had never received antiretroviral therapy. Her medications included trimethoprim/sulfamethoxazole, paroxetine, nifedipine, furosemide, and methadone.

The patient was lethargic (oral temperature 40.3°C, blood pressure 82/45 mm Hg, pulse rate 100 beats per minute, and respiratory rate 24 breaths per minute). Physical examination showed meningismus, bilateral conjunctival hemorrhages, diffuse petechiae, and tender palpable purpura on the lower extremities. Neurologic examination was nonfocal. Initial laboratory findings were as follows: hematocrit, 46%; platelet count, 69 x $10^9$/L; leukocyte count, 6.0 x $10^9$/L; creatinine, 1.6 mg/dL; aspartate aminotransferase, 61 U/L; alanine aminotransferase, 28 U/L; total bilirubin, 0.8 mg/L; alkaline phosphatase, 96 U/L; prothrombin time/international normalized ratio,14.1 seconds/1.3; partial thromboplastin time, 29.4 seconds. Urinalysis showed 14 red blood cells and 28 leukocytes per high-power field, no casts, and 3+ proteinuria. A later urinalysis showed that both the proteinuria and cellular elements had resolved. No schistocytes were visible on peripheral blood smear. A skin lesion biopsy performed in the emergency room revealed a thrombotic vasculopathy without evidence of rickettsiae (by direct immunoflourescence) or other microorganisms.

Because of concern for possible bacterial meningitis, the patient was immediately given 2 g ceftriaxone intravenously. After computed tomography of the brain revealed no acute intracranial process, lumbar puncture was performed. The cerebrospinal fluid was cloudy, with 7,675 nucleated cells/mm (92% neutrophils and 3% band forms). Protein was 382 mg/dL, and glucose was less than 20 mg/dL. Gram stain showed 3+ gram-negative diplococci. Cultures of the cerebrospinal fluid and blood yielded pure growth of serogroup Y *Neisseria meningitidis.*

The patient received a 10-day course of intravenous ceftriaxone, 2 g every 12 hrs. Whole hemolytic complement (CH50) drawn on hospital

Address for correspondence: Vance G. Fowler, Jr., Box 3281, Division of Infectious Diseases, Duke University Medical Center, Durham, NC 27710 USA; fax: 919-684-8902; e-mail: Fowle003@mc.duke.edu.

day 5 was 13 units (23-52 units). Other complement assays included a C3 value of 105 mg/dL (86-208 mg/dL) and a C4 value of 8 mg/dL (13-47 mg/dL. Results of a cryoglobulin screen were positive. Computed tomography of the abdomen revealed nodular liver and splenomegaly consistent with cirrhosis. The patient was given meningococcal vaccine near the end of her hospital course and was discharged with no sequelae. On follow-up with her primary physician, she had no evidence of complications from the meningococcemia. A repeat CH50 drawn 6 months after hospitalization was <10 units (23-52 units).

### Conclusions

To our knowledge this is the first reported case of disseminated meningococcemia in a patient coinfected with HIV and HCV. Because coinfected patients constitute an increasing percentage of patients infected with HIV (2), several features of this case bear emphasis.

First, hepatic dysfunction from conditions such as HCV is an important risk factor for meningococcal disease (3). This increased risk is likely due to decreased hepatic synthesis of complement (3). Because hypocomplementemia occurs commonly in patients infected with HCV, particularly when cirrhosis or cryoglobulinemia is present (4), these patients are at increased risk for meningococcal infection (3). Patients who are coinfected with HIV and HCV may be at even greater risk for meningococcal infection because of accelerated hepatic destruction. For example, patients coinfected with HIV and HCV have a higher progression to hepatic fibrosis (1) and a 3.5-fold increase in hepatic cirrhosis (2), when compared to patients with HCV alone. Given that up to 9% of HIV-infected patients may be coinfected with HCV (2), a group of patients with potentially increased susceptibility to meningococcal infection may be emerging.

Second, unlike the interaction between HIV and *S. pneumoniae*, *Salmonella*, and other recognized bacterial opportunistic pathogens, the interaction between HIV and meningococcus is unclear. Fewer than 50 cases of meningococcal infections in HIV-infected patients have been reported in MEDLINE (5-14). These reports present conflicting results on the impact of HIV infection on the risk for meningococcal disease. For example, while one recent prospective cohort study reported an increased risk for meningococcal disease (relative risk 23.8, confidence interval 7.4-76.7; p <0.001) in HIV-infected patients in the Atlanta metropolitan area (12), a case-control study in Africa showed no link between HIV infection and epidemic meningococcal disease (14). Despite the low rate of reported infection, asymptomatic colonization with meningococcus occurs in as many as 15%-23% of tested cohorts of both HIV-infected patients (15) and healthy young adults (16).

There are limitations to our report. Because our patient had several pneumococcal infections before the meningococcemia, immunologic defects other than hypocomplementemia (such as advanced HIV) may have contributed to susceptibility to both of these bacterial pathogens. Only limited data may be inferred from a single case. For example, assuming that the overall rate of meningococcal disease in the United States is 1/100,000, that 1% of the U.S. population is HIV positive, and that 9% of these patients are coinfected with HCV, only two to three cases of meningococcal infection would be expected to occur among coinfected patients if no additional risk for meningococcal infection were present. Because the true incidence of meningococcal infection among coinfected patients is unknown, future cohort studies will have to establish the impact of coinfection with HIV and HCV on the risk for meningococcal infection.

This study was supported by National Institutes of Health grant AI-01647.

Dr. Nelson is a third-year resident in the Department of Internal Medicine at Duke University Medical Center, Durham, North Carolina. He has a DVM degree from the School of Veterinary Medicine at University of California Davis. His research interests include pulmonary and critical care medicine as well as zoonotic diseases.

### References

1. Benhamou Y, Bochet M, DiMartino V, Charlotte F, Azria F, Coutellier A, et al. Liver fibrosis progression in human immunodeficiency virus and hepatitis C virus coinfected patients. Hepatology 1999;30:1054-8.
2. Zylberberg H, Pol S. Reciprocal interactions between human immunodeficiency virus and hepatitis C virus infections. Clin Infect Dis 1996;23:1117-25.
3. Ellison RT, Mason SR, Kohler PF, Curd JG, Reller LB. Meningococcemia and acquired complement deficiency. Association in patients with hepatic failure. Arch Intern Med 1986;146:1539-40.

4. Weiner SM, Berg T, Berthold H, Weber S, Peters T, Blum, et al. A clinical and virological study of hepatitis C virus-related cryoglobulinemia in Germany. J Hepatol 1998;29:375-84.
5. Aguado JM, Vada J, Zuniga M. Meningococcemia: an undescribed cause of community-acquired bacteremia in patients with acquired immunodeficiency syndrome (AIDS) and AIDS-related complex. Am J Med 1990;88:314.
6. Assier H, Chosidow O, Rekacewicz I, Lionnet F, Pipau FG, Riou JY, et al. Chronic meningococcemia in acquired immunodeficiency infection. J Am Acad Dermatol 1993;29:793-4.
7. Garcia-Lechuz JM, Alcala L, Gijon P, Juan R, Bouza E. Primary meningococcal conjunctivitis in a human immunodeficiency virus-infected adult. Clin Infect Dis 1998;27:1556-7.
8. Gilks CF, Brindle RJ, Otieno LS, Simani PM, Newnham RS, Bhatt SM, et al. Life-threatening bacteraemia in HIV-1 seropositive adults admitted to hospital in Nairobi, Kenya. Lancet 1990;336:545-9.
9. Kipp W, Kamugisha J, Rehle T. Meningococcal meningitis and HIV infection: results from a case-control study in western Uganda. AIDS 1992;6:1557-8.
10. Morla N, Guibourdenche M, Riou JY. *Neisseria* spp. and AIDS. J Clin Microbiol 1992;30:2290-4.
11. Nitta AT, Douglas JM, Arakere G, Ebens JB. Disseminated meningococcal infection in HIV-seropositive patients. AIDS 1993;7:87-90.
12. Stephens DS, Hajjeh RA, Baughman WS, Harvey RC, Wenger JD, et al. Sporadic meningococcal disease in adults: results of a 5-year population-based study. Ann Intern Med 1995;123:937-40.
13. Winters RA, Helfgott D, Storey-Johnson C, Murray HW. Human immunodeficiency virus infection and bacteremic meningococcal pneumonia. J Infect Dis 1991;163:1390.
14. Pinner RW, Onyango F, Perkins BA, Mirza NB, Ngacha DM, Reeves M, et al. Epidemic meningococcal disease in Nairobi, Kenya, 1989. J Infect Dis 1992;166:359-64.
15. Carlin EM, Hannan M, Walsh J, Talboys C, Shah D, Flynn R, et al. Nasopharyngeal flora in HIV seropositive men who have sex with men. Genitourinary Medicine 1997;73:477-80.
16. Neal KR, Nguyen-Van-Tam JS, Jeffrey N, Slack RC, Madeley RJ, et al. Changing carriage rate of *Neisseria meningitidis* among university students during the first week of term: cross sectional study. Br Med J 2000;320:846-9.
